# Long Non-Coding RNAs and Alzheimer’s Disease: Towards Personalized Diagnosis

**DOI:** 10.3390/ijms25147641

**Published:** 2024-07-11

**Authors:** Maria I. Mosquera-Heredia, Oscar M. Vidal, Luis C. Morales, Carlos Silvera-Redondo, Ernesto Barceló, Ricardo Allegri, Mauricio Arcos-Burgos, Jorge I. Vélez, Pilar Garavito-Galofre

**Affiliations:** 1 Department of Medicine, Universidad del Norte, Barranquilla 081007, Colombia; mosquerai@uninorte.edu.co (M.I.M.-H.); oorjuela@uninorte.edu.co (O.M.V.); burbanoc@uninorte.edu.co (L.C.M.); csilvera@uninorte.edu.co (C.S.-R.); 2 Instituto Colombiano de Neuropedagogía, Barranquilla 080020, Colombia; erbarcelo@yahoo.com; 3 Department of Health Sciences, Universidad de La Costa, Barranquilla 080002, Colombia; 4 Grupo Internacional de Investigación Neuro-Conductual (GIINCO), Universidad de La Costa, Barranquilla 080002, Colombia; 5 Institute for Neurological Research FLENI, Montañeses 2325, Buenos Aires C1428AQK, Argentina; rallegri@fleni.org.ar; 6 Grupo de Investigación en Psiquiatría (GIPSI), Departamento de Psiquiatría, Instituto de Investigaciones Médicas, Facultad de Medicina, Universidad de Antioquia, Medellin 050010, Colombia; mauricio.arcos@udea.edu.co; 7 Department of Industrial Engineering, Universidad del Norte, Barranquilla 081007, Colombia

**Keywords:** Alzheimer’s disease, exosomes, long non-coding RNA, machine learning, personalized medicine

## Abstract

Alzheimer’s disease (AD), a neurodegenerative disorder characterized by progressive cognitive decline, is the most common form of dementia. Currently, there is no single test that can diagnose AD, especially in understudied populations and developing countries. Instead, diagnosis is based on a combination of medical history, physical examination, cognitive testing, and brain imaging. Exosomes are extracellular nanovesicles, primarily composed of RNA, that participate in physiological processes related to AD pathogenesis such as cell proliferation, immune response, and neuronal and cardiovascular function. However, the identification and understanding of the potential role of long non-coding RNAs (lncRNAs) in AD diagnosis remain largely unexplored. Here, we clinically, cognitively, and genetically characterized a sample of 15 individuals diagnosed with AD (cases) and 15 controls from Barranquilla, Colombia. Advanced bioinformatics, analytics and Machine Learning (ML) techniques were used to identify lncRNAs differentially expressed between cases and controls. The expression of 28,909 lncRNAs was quantified. Of these, 18 were found to be differentially expressed and harbored in pivotal genes related to AD. Two lncRNAs, ENST00000608936 and ENST00000433747, show promise as diagnostic markers for AD, with ML models achieving > 95% sensitivity, specificity, and accuracy in both the training and testing datasets. These findings suggest that the expression profiles of lncRNAs could significantly contribute to advancing personalized AD diagnosis in this community, offering promising avenues for early detection and follow-up.

## 1. Introduction

Alzheimer’s disease (AD) is the predominant form of dementia globally, representing a significant public health concern due to its prevalence as a leading cause of disability and dependency in the elderly. According to the World Health Organization (WHO), AD accounts for 60–70% of reported cases of dementia and has significant economic impacts in terms of direct medical costs, social care, and informal care, the latter represented by the loss of income of caregivers [1]. Moreover, the condition exerts a profound physical, psychological, and social toll on both caregivers and families, underscoring the urgent need for comprehensive understanding and effective interventions in addressing this complex and challenging neurodegenerative disorder [1,2].

Over the last three decades, significant research efforts have been directed towards elucidating the molecular pathophysiology of AD [3,4]. This understanding serves as a foundation for developing novel therapeutic and diagnostic applications, ultimately mitigating the impact of this debilitating disorder on patients and their caregivers [5,6]. Unfortunately, there is no single test that can diagnose AD, especially in understudied populations and developing countries. Instead, diagnosis is based on a combination of medical history, physical examination, cognitive testing, and brain imaging [7,8]. By advancing our knowledge of the underlying mechanisms and introducing innovative interventions and diagnostic tools, we can work towards improving the quality of life for individuals affected by AD, including both patients and their support networks [9,10]. 

Exosomes, extracellular nanovesicles originating from endocytic pathways, have emerged as a novel mechanism for intercellular molecular transport [11]. These vesicles contain a diverse array of components including proteins, lipids, coding RNA, and non-coding RNA (ncRNA) that can exert either beneficial or detrimental effects upon interaction with target cells, depending on the context [12]. Exploring the role of exosomes in neurodegenerative disorders like Alzheimer’s disease (AD) not only enhances our comprehension of cellular communication underlying both normal and pathological processes in the brain but also sheds light on their involvement in critical functions such as synaptic plasticity, myelin membrane biogenesis regulation, and localized transfer of proteins or nucleic acids to specialized structures like neurons [13,14,15]. Furthermore, exosomes may create an environment conducive to amyloid fibril formation, thereby significantly influencing the pathogenesis of AD [14]. Examination of blood exosome contents holds significant importance, especially given the formidable protection of the central nervous system (CNS) and its limited accessibility. Therefore, it is possible to obtain information about its cells from exosomes that cross the blood–brain barrier [16], which means that no invasive intervention is required for its analysis [17]. 

ncRNAs represent an important part of the genome and regulate the expression of genes that may be involved in AD [18]. In 2001, the Human Genome Project (HGP) revealed that coding regions only represent approximately 2% of the entire genome [19]. Subsequently, the Encyclopedia of DNA Elements (ENCODE) project concluded that about 80% of the human genome is transcribed as non-protein-coding elements. Although it was initially considered “junk DNA”, it was later determined that a large portion of the non-coding regions were functional [20]. These ncRNAs are now known to play an important role in the regulation of gene expression, many of which are involved in disease pathogenesis [21]. Therefore, its study emerges as a novel option to understand it.

In AD, research in this field is just beginning [22,23]. In particular, microRNAs (miRNAs) are the most studied ncRNAs [22]. However, long ncRNAs (lncRNAs), which are transcripts over 200 nucleotides in length with no apparent protein-coding capacity, have received increasing attention and are expected to be novel epigenetic regulators of gene expression at the transcriptional and post-transcriptional levels [24]. 

lncRNAs are widely expressed in the brain and affect the proliferation, survival, metabolism, and differentiation of neuronal cells and are, therefore, considered to contribute to the pathogenesis of AD [25]. Compelling evidence has shown that lncRNAs are aberrantly expressed in AD progression and modulate beta amyloid beta (Aβ) peptide formation, Tau hyperphosphorylation, neuroinflammation, and neuronal apoptosis [26,27]. However, we still need to identify new lncRNAs involved, analyze their differential expression, and clarify how they participate in the pathogenic pathways of AD. This new information would allow us to establish lncRNAs as future biomarkers or therapeutic targets for this form of dementia.

As part of a large collaborative effort to elucidate the genetic landscape of genomic variation that confers susceptibility to AD, since 2020, we have clinically, cognitively, and neuropsychologically assesses a sample of individuals with sporadic AD (cases) and healthy controls from Barranquilla, Colombia. In this study, we quantified the expression of 29,809 lncRNAs using microarrays and employed advanced bioinformatics, data analytics, and Machine Learning (ML) techniques to identify lncRNAs differentially expressed between cases and controls and evaluate their potential to determine the diagnosis of AD. Our working hypothesis is that, since many of the risk variants associated with AD are found in non-coding or intergenic regions [18], lncRNAs could be promising non-invasive and reliable novel diagnostic markers for AD in this population [28]. 

## 2. Results

### 2.1. Subjects

We studied 15 individuals with a positive diagnosis of AD and 15 healthy controls. Table 1 summarizes the demographic characteristics of all participants. 

### 2.2. Differentially Expressed lncRNAs

**Figure 1:**
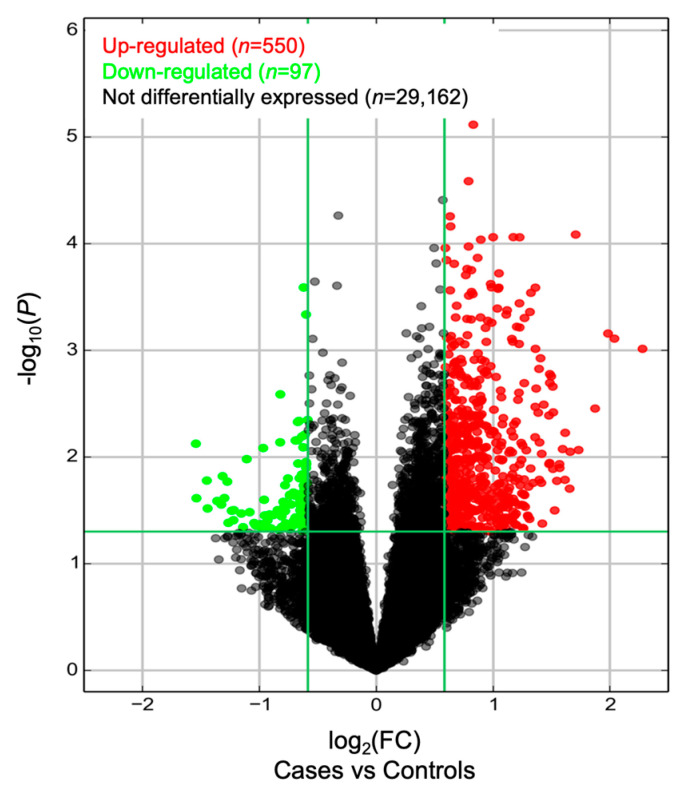
Volcano plot for the lncRNA expression values quantified in individuals with AD and healthy controls.

In this study, the expression of 29,809 lncRNAs was quantified. A total of 647 were found to be differentially expressed between subjects with AD and those in the control group, according to the company’s default settings (|FC| > 0.5; Figure 1). Of these, 550 were found to be upregulated and 97 downregulated between the comparison groups.

Table 2 shows the top upregulated lncRNAs in our sample of individuals with AD. Of these, the top-10 lncRNAs with a FC ≥ 1.5 are harbored in the *TMEM186*, *PROX1-AS1*, *AC109635.2*, *LINC02043*, *AC022031.2*, *AP003175.1*, *POT1-AS1*, *AL020993.1*, *XLOC_012031,* and *CATG00000032665* genes.

Table 3 shows the top downregulated lncRNAs in our sample of individuals with AD. Of these, the top-10 lncRNAs with a FC ≥ 1.5 are harbored in the *AC073529.1*, *C5orf64*, *G090124*, *TAB2-AS1*, *AC117382.2*, *G014791*, *AC007342.1*, *HTR2A-AS1*, *PTBP2,* and *IL7* genes. Interestingly, most of these differentially expressed lncRNAs, either up- or downregulated, are intergenic and have a length >215 nt.

For further in silico analyses, the 18 differentially expressed lncRNA (10 up- and 8 down-regulated in the AD group compared to healthy controls) were selected according to (1) the *p*-value and the highest FC and (2) the functional relevance with AD of the lncRNA-associated genes (Table 4). These lncRNAs participate in key biological processes related to AD pathogenicity, including neurogenesis and cell differentiation, proteostasis of Aβ-peptide, neuroinflammation, neurite growth, synaptic plasticity, and apoptosis (Table 5).

**Figure 2:**
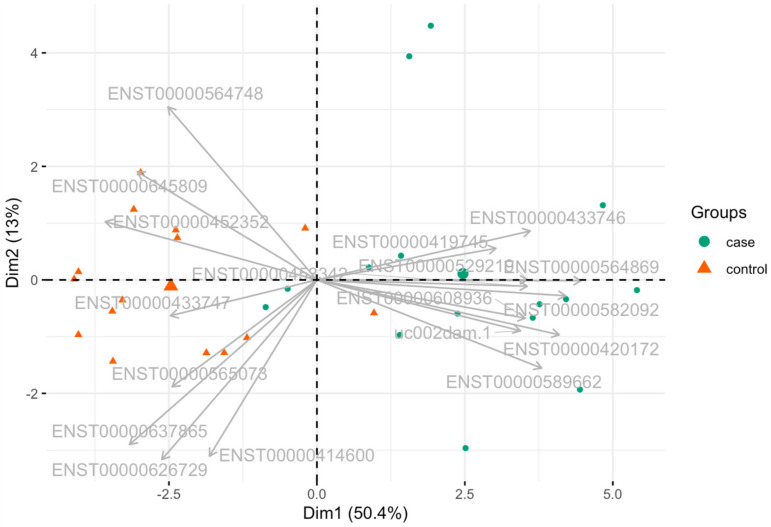
Biplot based on the expression levels of 18 lncRNAs differentially expressed.

Based on the 18 lncRNAs selected (Table 4), Principal Component Analysis (PCA) was applied to visualize the joint distribution of all individuals in a multidimensional space and evaluate the potential of these lncRNAs to differentiate individuals with AD from healthy controls. Figure 2 shows the biplot for the first two principal components, which explain ~64% of the total variance, along with the individuals (green [cases] and orange [controls] dots) and the direction of the selected lncRNAs. Notably, control individuals are predominantly clustered in the II and III quadrants, whereas individuals diagnosed with AD are primarily located in the I and IV quadrants. This suggests that the selected lncRNAs have promising potential for developing ML-based predictive models for AD diagnosis.

### 2.3. Protein–Protein Interactions (PPIs) between lncRNA-Associated Genes

Analysis of protein–protein interaction (PPI) revealed an established network between the lncRNAs *AC022031.2* and *CAS6-AS1*, which is potentially associated with the *CAS6* gene. When STRING V11.5 (https://string-db.org/; accessed on 5 July 2023) was used with all proteins coded by the 18 lncRNA-associated genes, we found statistically significance evidence (*P* < 0.05 and an average clustering coefficient [ACC] > 0.7), as well as enrichment in biological pathways and molecular functions related to AD in the PPI of proteins PMM2, PROX1, SNX8, SMAD2, GAS6, POT1, SRCIN1, CIITA, SS18, ABI2, TAB2, RPGR, RBL2, HTR2A, and SOX9. Results are shown in Table 6.

### 2.4. Biological Relatedness between lncRNA-Associated Genes and AD-Associated Genes

We used the Human Genome Connectome Database (HGC; https://hgc.rockefeller.edu/; accessed on 5 July 2023) to quantify the biological relatedness between the top-10 AD-associated genes and those identified via lncRNA. A nominal statistically significant biological relatedness was found between *GAS6*, the target gene of the *AC022031.2—CAS6-AS1* network, and the AD-associated genes *CLU*, *APOE*, *ABCA7,* and *CD2AP* (*P* < 0.05, Table 7). Likewise, the gene *SMAD2*, the possible target gene of *LINC02043*, is biologically related to *APOE*, *CLU*, *BIN1*, *CD33,* and *ABCA7* (*p* < 0.05, Table 7), while *HTR2A*, the target gene of the *HTR2A-AS1* lncRNA, is biologically related to *CLU* and *ABCA7*. Similarly, *RBL2* and *CIITA* are biologically related to *CD33*, while *MUC2*, *SRCIN1*, *SS18,* and *POT1* are biologically related to *CR1*, *CD2AP*, *BIN1,* and *PICALM*, respectively.

### 2.5. ML-Based Diagnostic Assessment 

**Figure 3:**
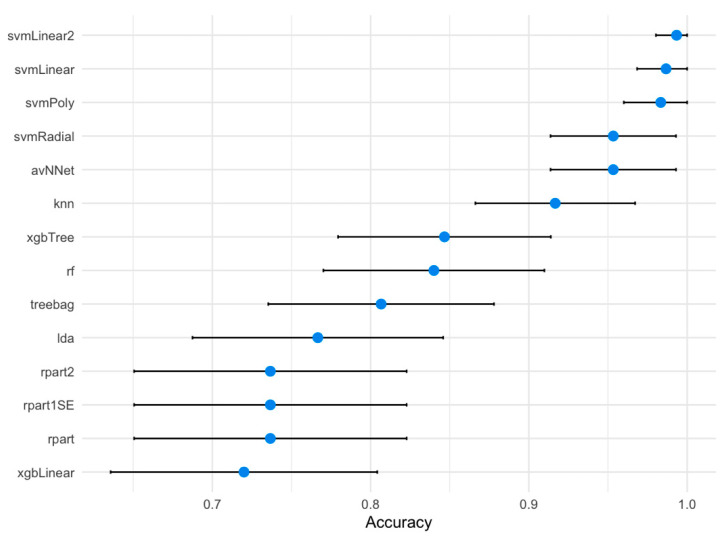
Accuracy and 95% confidence intervals for predicting AD diagnosis based on selected lncRNAs.

We evaluated the performance of 14 distinct Machine Learning (ML) algorithms in predicting AD diagnosis based on the 18 previously selected lncRNAs (Table 4 and Table 5). The balanced accuracy of these ML algorithms is shown in Figure 3. Notably, the svmLinear2, svmLinear, and svmPoly algorithms demonstrated exceptional performance, with accuracy values exceeding 98% in the training dataset. Among these, the svmLinear2 algorithm emerged as the top performer, showcasing its potential in accurately diagnosing AD using lncRNA-based data.

**Figure 4:**
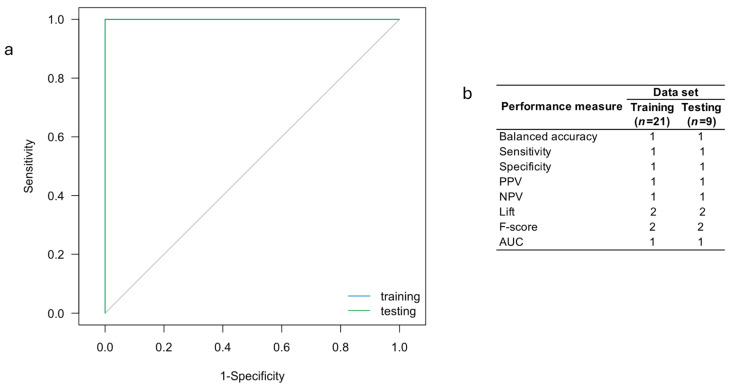
(**a**) ROC curve and (**b**) performance measures for the svmLinear2 ML model using the expression levels of the 18 lncRNAs-based for predicting AD diagnosis.

Analysis of the ROC curve for the svmLinear2 ML algorithm indicates that this model provides a high ability to discriminate individuals with AD from healthy controls (AUC > 0.9; Figure 4). This algorithm was found to have competitive values for sensitivity, specificity, accuracy, PPV, NPV, and lift (Figure 4b), which strongly suggests that this algorithm is a potential tool for early diagnosis of AD in the clinical setting.

**Figure 5:**
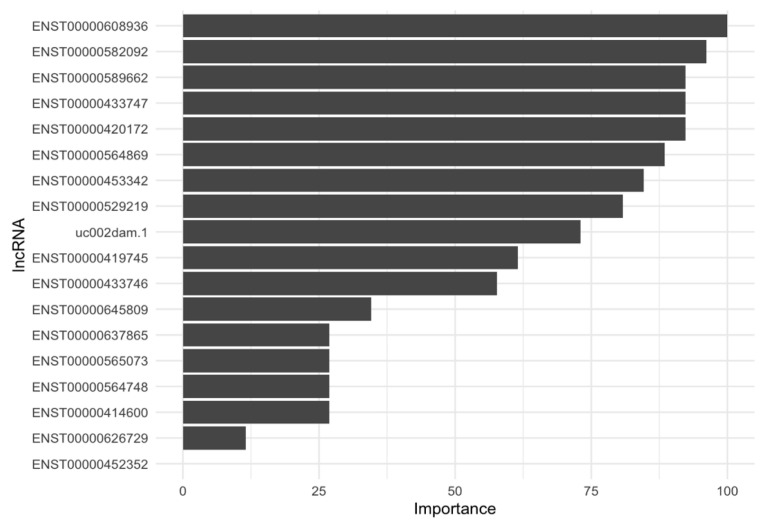
Importance of selected lncRNAs for predicting AD diagnosis with the svmLinear2 ML algorithm.

Variable importance analyses reveal that combining the expression of the 18 selected lncRNAs with demographic variables, such as gender, age, and educational level, enhances the predictive power for AD diagnosis; two lncRNAs (ENST00000608936 harbored in *PROX1-AS1* and ENST00000582092 harbored in *SS18*) are the most important predictors (Figure 5). These findings emphasize the significant role that these specific lncRNAs play in AD diagnosis, even when accounting for demographic factors that may influence disease risk and progression.

As complementary analyses, we utilized the One-Rule (OneR) [30] ML algorithm to identify the most relevant lncRNA for predicting AD diagnosis in our sample. Our results indicate that the lncRNA *PROX1-AS1* (ENST00000608936) is the main driver of AD diagnosis in our sample (Table 8). Interestingly, using the svmLinear2 ML algorithm with this lncRNA showed poor predictive power (not reported).

**Figure 6:**
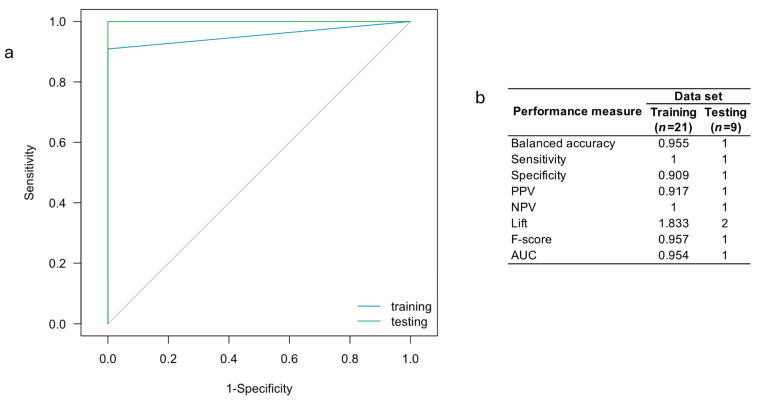
(**a**) ROC curve and (**b**) performance measures for the svmLinear2 ML model using the expression levels of the OneR-derived lncRNAs for predicting AD diagnosis.

However, by combining *PROX1-AS1* (ENST00000608936) and *AC073529.1* (ENST00000433747), we observed exceptional performance for predicting AD diagnosis in our sample (Figure 6).

## 3. Discussion

Alzheimer’s disease (AD) is the most common type of age-related dementia worldwide [31], characterized by the development of extracellular plaques formed by amyloid beta (Aβ) peptides and neurofibrillary tangles composed of hyperphosphorylated Tau protein (p-Tau). However, in clinical trials, reducing the production of Aβ peptides in the brain did not halt cognitive decline or improve the quality of life of AD patients. Hence, other pathogenic mechanisms have been proposed, suggesting a multifactorial nature of AD [32]. Although research studies have identified genes associated with sporadic AD [33], understanding the regulation of gene expression would allow us to better comprehend the pathogenic network of the disease.

Non-coding RNAs (ncRNAs) have been shown to play an important role in the regulation of gene expression, many of which are involved in the pathogenesis of disease. [21]. In this study, we characterized the differential expression profile of long ncRNA (lncRNA) contained in circulating exosomes in a group of individuals with AD and a control group to clarify how they participate in the pathogenic pathways of the disease. This information would help to establish lncRNAs as future biomarkers or therapeutic targets for AD.

We identified a total of 647 lncRNAs to be differentially expressed between the comparison groups according to the company’s default settings (Figure 1). Of these, 550 were upregulated and 97 were downregulated in patients with AD. Among the top 20 lncRNAs differentially expressed within each group, only *PCA3* has previously been associated with AD [34]. Through in silico analysis, we predicted that selected lncRNAs (Table 4) interact with possible target genes and impact their participation in different pathways that integrate the pathogenic network of AD (i.e., neurogenesis, cell differentiation, proteostasis of Aβ peptide and p-Tau, neuroinflammation, chromatin remodeling, neurite outgrowth, synaptic plasticity, apoptosis, and cell cycle control) and situations favoring AD development (i.e., depression, ciliopathies, and alteration of the intestinal barrier) (Table 5). Based on the selected lncRNAs, we proposed and validated different ML-based predictive model for AD diagnosis (Figure 3, Figure 4 and Figure 6). Our results suggest that some of these lncRNAs lead to remarkable predictive power to distinguish individuals and those with AD, showing promise in the clinical setting.

The IPP network of the *PMM2* gene, which codes for the phosphomannomutase 2 protein (*P* = 0.00056) and guanosine diphosphate mannose precursor (GDP-manosa) (*P* = 1.62 × 10^−8^), participates in Aβ-peptide proteases in the brain. GDP-mannose is required for the synthesis of dolichol phosphate-mannose involved in the N-glycosylation of proteins [35]. An increase in dolichol phosphate (without addition of the oligosaccharide) has been demonstrated in the brain of subjects with AD [36], which could lead to the accumulation of Aβ peptide [36], since it is related to the decrease in P-glycoprotein (P-gp), which participates as an ejector pump of this peptide across the blood–brain barrier (BBB) [37]. Other IPP networks associated with Aβ proteasome include networks with proteins encoded by the *SNX8*, *SMAD2*, *GAS6*, *TAB2,* and *SOX9* genes.

SNX8 is part of the retromeric protein complex (*P* = 8.15 × 10^−17^), which is an essential part of the endosomal system (*P* = 2.31 × 10^−9^) [38], participating in the redistribution of APP from the Golgi apparatus to the cytoplasmic membrane, where soluble fragments are generated through cleavage of this protein by α-secretases [39]. SMAD2, a protein that participates in the signaling pathway of transforming growth factor beta 1 (TGF-β1) (*P =* 1.92 × 10^−9^), provides a stimulus for microglia to achieve phagocytosis of Aβ peptides [40]. Likewise, the ligands of the TAM receptors GAS6 and PROTS bind through their N-terminal region to phosphatidylserine (*P =* 9.58 × 10^−5^) present in phagocytic targets such as apoptotic bodies (*P =* 0.0037) or in the Aβ peptides. In turn, they bind through their C-terminal region to TAM receptors present in microglial phagocytes [41]. TAB2 is required for the canonical activation of the Nuclear Factor Kappa B (NF-κB) signaling pathway, which promotes the transcription of genes such as *BACE1* and *SOX9*, among others [42]. The increase in the transcription of *BACE1* leads to increased APP processing via the amyloidogenic pathway [43], while *SOX9* may play a role in Aβ deposition in astrocytes and responding to the presence of amyloid plaque in the brain [44,45].

While microglia activation is necessary for Aβ-peptide phagocytosis, their chronic overactivation promotes migration and clustering of these cells into amyloid plaques, thereby constantly releasing toxins, which actively contribute to the progressive neurodegeneration characteristic of AD [46]. In this regard, the modulation exerted by TGF-β1-Smad2/Smad3, which promotes Aβ peptide clearance by microglial phagocytosis, becomes necessary. However, it has also been shown to regulate microglial migration toward Aβ plaques with consequent modulation of the inflammatory response [40,47]. Likewise, the GAS6 IPP network involving TAM receptors (Tyro3, Axl, and Mer) and other ligands participates in the inhibition of the propagation of pro-inflammatory signals resulting from Toll-like receptor (TLR) stimulation and induces the release of anti-inflammatory molecules such as interleukin-10 (IL-10) and transforming growth factor β (TGF-β) [48,49].

In contrast, the IPP network of TAB2 and SOX9 and the IPP network of CIITA promote the inflammatory response in the brain, since NF-κB (activated by the MAP3K7/TAK1/TAB2/TAB3/TAB2/TAB3 complex) promotes the inflammatory response in the brain and the transcription of coding genes for inflammatory cytokines (such as IL-1β, IL-6, IL-12, and TNF [50]) and SOX9, which could play an important role in the activation of astrocytes and release of chondroitin sulfate pro-teoglycans that contribute to glial scar formation in AD individuals [51]. The CIITA IPP network regulates the biosynthetic process of Major Histocompatibility Complex (MHC) class II on the surface of innate immune cells in the brain (*p =* 0.0170). This favors the presentation of Aβ peptide as an antigen (*p =* 6.02 × 10^−13^), further enhancing the pro-inflammatory phenotype of microglia.

On the other hand, the IPP networks of SMAD2 and GAS6, and that of PROX1, participate in neurogenesis and cell differentiation. In this case, TGFβ/activin (*P =* 1.52 × 10^−6^)-regulated R-Smads (*P =* 4.48 × 10^−6^), Smad2 and Smad3, target specific genes for transcriptional regulation (*P =* 4.27 × 10^−5^), thereby promoting neurogenesis from neural stem cells in the subgranular zone of the hippocampus. Newly generated neurons migrate steadily to the granule cell layer and integrate into existing neural circuits [52,53]. In turn, the binding of GAS6 and PROTS to TAM receptors promotes neural stem cell proliferation, differentiation into mature neurons, and migration and cell survival through the regulation of neurotrophin expression, especially nerve growth factor expression (*P =* 0.0028) [54,55], while the *PROX1* gene, whose transcription is induced by activation of the Wnt/β-catenin pathway, is required for proper differentiation of granule cells (*P =* 0.0281) during embryonic and adult neurogenesis in the hippocampus, but not for the maintenance of mature granule cells [56].

We found that the ABI2 network, which is part of the Wiskott–Aldrich syndrome protein complex and is a protein homologous to verprolin (WAVE) [57], participates in the control of neurite outgrowth by activating the Arp2/3 complex (*P =* 3.77 × 10^−16^) to promote the formation of new actin filaments (*P =* 5.40 × 10^−12^), which allow the creation of branched networks that constitute the morphogenesis of the dendrite. Similarly, p140Cap can maintain dendritic spine (DE) morphology by locally regulating actin polymerization through interaction with postsynaptic density components, for example, by directly inhibiting Src kinase activation (*P =* 0.0493) and binding to the Citron-N protein (*P =* 0.0493) [58].

The SRCIN1 IPP network was also found to be involved in the synaptic transmission process (*P =* 0.0236). According to GO annotations on cellular components, the proteins that interact with p140Cap are in the membranes of synaptic vesicles (*p =* 0.0066). p140Cap controls synaptic plasticity in differentiated neurons and regulates GABAergic synaptogenesis and the development of hippocampal inhibitory circuits [59]. 

Pathological alterations in AD involve an abnormal apoptotic cascade in susceptible brain regions. Dysregulated apoptosis ends with abnormal neuronal loss, which is considered as a primary event that may precede the other events of AD progression [60]. In this study, IPP networks suggest that proteins encoded by RBL2, TAB2, SOX9, and GAS6 genes are involved in apoptosis.

According to KEGG analysis, the RBL2 IPP network participates in the cell cycle (*P =* 4.31 × 10^−19^) and senescence (*P =* 1.84 × 10^−18^) due to their biological functions related to transcription regulation (*P =* 0.0059) and DNA damage response (*P =* 3.12 × 10^−5^). Several Rb family proteins, including pRb and p130 (RBL2), have been shown to suppress the cell cycle by controlling the G1/S transition of the mitotic cell cycle (*p* = 0.00029) in differentiated neurons. However, in neurodegenerative diseases such as EAE, this process is disrupted when Rb expression decreases or dissociates from EF2 transcription factors, forcing mature neurons to re-enter the cell cycle [55]. The NF-κB signaling pathway, activated through the formation of a complex between MAP3K7/TAK1 kinase and TAB2 [42], has also been linked to apoptosis, since NF-κB modulates the expression of some protein-coding genes such as p53, c-Myc, cyclin D1, Bcl, and BAX [61]. Thus, under-expression of the *SOX9* gene through the NF-κB signaling pathway increases the expression of the anti-apoptotic Bcl-2 protein and decreases the expression of the pro-apoptotic Bax protein [44]. In contrast, the GAS6 IPP network exerts a negative regulation of the dendritic cell apoptotic process (*P =* 0.0037) by counteracting the detrimental intracellular Ca^2+^ increase induced by Aβ and favoring the uptake of apoptotic bodies by macrophages [62].

Different epigenetic mechanisms, including chromatin remodeling, may be altered in neurodegenerative disorders such as AD [63]. SS18 is a component of the GBAF subcomplex belonging to the SWI/SNF (SWItch/Sucrose Non-Fermentable) family (*P* = 5.42 × 10^−29^), which remodels chromatin through histone acetylation (*P =* 0.0142). Alteration of this process is involved in cell differentiation, apoptosis, inflammatory reaction, neuronal plasticity, and synaptogenesis [64]; these pathways integrate the metabolic network of AD.

Based on emerging evidence indicating a correlation between these processes and the pathogenesis of AD, three potential target genes are postulated to be involved in the development of depression, ciliopathies, and intestinal barrier disruption [65,66,67]. Thus, the possible involvement of the HTR2A PPI network in the development of depression was identified. This network participates in the serotonin receptor signaling pathway (*P =* 0.00082), which regulates hippocampal activity under physiological conditions [68]. Its alteration has been shown to be related to mood disorders such as anxiety and depression, learning deficits, memory impairment, and, consequently, to AD [69]. The *ERICH3* gene, for which no PPI network could be established, is associated with a negative response to the treatment of depression with selective serotonin reuptake inhibitors (SSRIs) in subjects with AD [70]. On the other hand, the RPGR IPP network participates in the assembly of non-motile cilia (*P =* 0.0014) and in the trafficking of ciliary proteins (*P =* 3.41 × 10^−12^), therefore, this network could be determinant for the correct functioning of cilia [71]. Alterations in these processes, collectively referred to as ciliopathies, have been associated with aging and age-related brain disorders [66]. Conversely, the MUC2 gene, for which an IPP network could not be established, is implicated in intestinal barrier integrity. This is significant as disruptions in intestinal permeability can lead to the translocation of microbial exudates, lipopolysaccharides (LPSs), and amyloid molecules into the bloodstream, facilitating their transport to the brain. Subsequently, this process triggers the activation of microglia and astrocytes, underscoring the intricate relationship between gut health and neuroinflammation in the context of AD [72]. In addition, deregulated MUC2 expression contributes to the alteration of the intestinal microbiota, which has been linked in recent years to the development of AD [73].

Current diagnostic approaches for AD are mainly based on neuropsychological assessments, brain imaging, and the detection of β amyloid-1-42 peptide (Aβ42), total Tau protein, and hyperphosphorylated Tau protein (p-Tau) in cerebrospinal fluid (CSF) [74,75]. However, these tests are expensive and invasive [76,77] and have low sensitivity and specificity [78]. Late diagnosis of AD in clinical settings has raised concerns, emphasizing the need for cost-effective and accessible blood biomarkers to enable early detection of AD, before significant brain damage occurs. Early diagnosis would allow patients to benefit more from available treatments, potentially slowing disease progression and improving outcomes [79].

ML algorithms have been used for the design of predictive algorithms for different diseases, including AD [80]. Jo et al. demonstrated, using meta-analysis, that studies that evaluated the role of artificial intelligence have accuracy values of 80% and 90% for predicting the evolution of MCI to AD and for classifying the type of AD, respectively [81]. Similarly, Rhodius-Master et al. studied how the combination of different neuropsychological tests could help identify individuals with MCI who were likely to develop clinical progression to AD [82]. Other researchers have included genetic and/or biochemical variables for disease prediction to improve the performance of these tools [83]. In Colombia, studies have identified ADAOO, AD status (early vs. late) and cognitive decline modifier genes in individuals with familial and sporadic AD (REFs). Furthermore, performance of different ML algorithms to predict ADAOO using demographic and genetic information has also been explored in individuals with familial and sporadic AD. In the latter, *GPR45*-rs35946826 and *MAGI3*-rs61742849 exhibit good predictive performance for the age of onset [6].

Several studies have integrated ML algorithms and lncRNAs signatures to predict prognosis, follow-up, and diagnosis in AD. For instance, a 2020 study assessed lncRNA expression profiles using data from the Gene Expression Omnibus (GEO) database. The authors found 47 differentially expressed lncRNAs between 57 AD samples and 57 healthy controls and ultimately applied a panel of 9 lncRNAs to train a ML model, which achieved an accuracy of 87.7% and 87.6% in the training and testing datasets, respectively, for classifying individuals with AD and healthy controls [84]. On the other hand, an SVM model using a signature of five lncRNAs predicts AD prognosis based on competing endogenous RNA networks and achieves an accuracy of 69% in a 10-fold cross-validation on 589 samples [85]. When this ML mode is tested on an independent dataset of 161 samples, the accuracy improved to 78.3% with sensitivity and specificity values of 77% and 79.7%, respectively [85]. More recently, a study developed an SVM-based model integrating lncRNA sequence and structure features to predict disease-related lncRNAs. This ML model achieves an F1 score of 76% in identifying lncRNAs associated with various diseases, including AD [86].

Our study evaluated the accuracy of 14 ML algorithms used to predict the diagnosis of sporadic AD from demographic data and the expression levels of the 18 selected lncRNAs (Figure 3). The svmLinear2, svmLinear, and svmPoly ML algorithms were found to provide accuracy rates > 98%. Among them, the svmLinear2 algorithm was the most accurate (Figure 3). Variable importance analysis (Table 8) revealed that the best predictors of AD in our sample were *PROX1-AS1* (ENST00000608936) and *SS18* (ENST00000582092). These results are in line with those of Sharma et al., who identified other lncRNAs with remarkable predictive performance to dissect individuals with AD from healthy controls based on microarray studies of the prefrontal cortex, medial temporal gyrus, hippocampus, and entorhinal cortex [87]. The data from this study, along with previous findings, suggest that lncRNAs are promising predictors of AD. The predictive model developed in this research represents a valuable clinical tool for anticipating the development of AD dementia. This model enables the identification of individuals at risk, allowing for the implementation of preventive strategies to delay the onset and/or progression of the disease in affected individuals.

## 4. Materials and Methods

### 4.1. Subjects

Our study is of the case/control type. Here, individuals diagnosed with sporadic AD, recruited at the Instituto Colombiano de Neuropedagogía (ICN), Barranquilla, Colombia, were considered as “cases”. A total of 15 individuals comprised this group. All individuals were >65 years of age, met the diagnostic criteria for AD according to the *Diagnostic Statistical Manual* (DSM) version V (DSM-V) [88] and had a Mini-Mental State Examination (MMSE) [89] between 0 and 18 points. Individuals within this group with familial AD (i.e., caused by a single-gene mutation and exhibiting early signs and symptoms), the presence of other neurological disease (i.e., cerebrovascular disease, frontotemporal dementia, dementia due to Lewy bodies, Parkinson’s disease, etc.), major psychiatric diseases (i.e., psychosis, schizophrenia, personality disorders, etc.), and psychoactive substance use or excessive alcohol consumption, as well as those unable to complete the clinical studies, were excluded.

On the other hand, 15 individuals comprised the “control” group, which corresponds to healthy non-family volunteers aged >65 years without suspected AD and a MMSE between 19 and 29 points. In this group, individuals with depression, mild cognitive impairment (MCI) or dementia, the presence of any neurological disorder, major psychiatric illnesses, and use of psychoactive substances or excessive alcohol consumption were excluded. We also excluded healthy participants unable to complete the clinical studies. 

The average age at study entry was 79.8 ± 8.7 years in all participants, 77.5 ± 8.5 years in the group of cases, and 82.1 ± 8.6 years in the healthy controls group. The AOO in individuals with AD was 72.1 ± 7.1 years. We found no statistically significant difference between groups in the age at study entry, weight, height, BMI, sex distribution, marital status, or educational level (Table 1). However, these groups differed in the MMSE and MoCA, with AD individuals exhibiting lower values than healthy controls (MMSE: 13.9 ± 9.5 vs. 25.2 ± 5.6, *P* = 0.001; MoCA: 5.5 ± 5.3 vs. 25.9 ± 3, *P* < 0.001).

### 4.2. Neuropsychological Assessment

After explaining to potential participants what the study consisted of and obtaining informed consent, the ICN team determined the eligibility of the candidates based on the results of the Montreal Cognitive Assessment (MoCA) [90] and the inclusion and exclusion criteria previously described. The MoCA is a screening test to identify possible cases of mild cognitive impairment (MCI), possible dementia, and healthy subjects. Subsequently, an exhaustive neuropsychological evaluation was performed, which included the following tests: Montreal Cognitive Assessment Test (MoCA) [90], Boston Denomination Test [91,92], Rey–Osterrieth Complex Figure [93], Rey Auditory Verbal Learning Test (RAVLT) [94], Trail Making Test (TMT) [95,96], Symbol Digit Modality Test (SDMT) [97], Stroop Color and Word Test [98], Token Test [99], Benton’s Visual Retention Test (BVRT) [100], Clock Drawing Test [101], Memory Scale subtest of the Wisconsin Card Testing Test [102], Geriatric Depression Screening Test [103], Global Deterioration Scale (GDS) [104], Barthel Functional Index [105], and Neuropsychiatric Inventory [106]. Finally, an electroencephalogram was performed for all participants.

Additional data for each participant such as age at the beginning of the study, sex, educational level, marital status, weight, and height were also recorded through the clinical history. In all participants diagnosed with AD, the age of onset (AOO) of the disease was defined as the age at onset of symptoms according to previous research [107,108]. AOO was determined during anamnesis with information provided by the patients or their relatives and by seeking confirmation from various sources, such as the neurologist’s assessment and neuropsychological evaluations. This strategy has been shown to be very accurate [109].

### 4.3. RNA Isolation

Once the clinical selection and characterization of the participants was completed, blood samples were collected for the isolation of circulating exosomes. A total of 6 mL of blood was obtained by conventional venipuncture in tubes, without additive, for each participant. The tubes were centrifuged at 4000 RPM for 10 min to obtain serum. The serum was centrifuged at 5000 RPM for 30 min to remove any remaining vesicles or detritus. The supernatant was subsequently transferred without disturbing the pellet to a new Eppendorf tube and placed on conventional ice until use.

A Total Exosome Isolation Reagent commercial kit (catalog #4478360, Thermo Fisher Scientific, Inc., Walthman, MA, USA) was used to isolate the exosomes following the manufacturer’s instructions with minor modifications standardized at the laboratories of Universidad del Norte, Barranquilla; 1000 µL of serum and 200 µL of the reagent were transferred to an Eppendorf tube. This mixture was homogenized in a vortex for 1 min to obtain a cloudy solution. This mixture was subsequently incubated at −20 °C for 30 min at rest and in a vertical position, taking care not to mix during or after incubation. Later, the mixture was centrifuged at 10,000 RPM for 30 min at room temperature. The supernatant was aspirated with a micropipette and discarded in order not to alter the pellet, since it contained the exosomes. The pellet was resuspended adding 200 μL of PBS 1× and, thus, ready for further studies. Resulting exosomes were characterized by scanning electron microscopy (SEM). For this purpose, exosomes were encapsulated with nanodiamond particles, and their sizes were meticulously confirmed, with measurements revealing a maximum diameter of 160 nm (Figure S1, Supplementary Material).

### 4.4. Exosomal RNA Extraction

For the extraction of RNA contained in exosomes, a technique based on the acid phenol–chloroform method was standardized in the laboratory of the Universidad del Norte. To resuspended exosomes (200 μL), we added 200 μL of a denaturing solution 2× and proceeded to vortex mix for 1 min. Then, this mixture was incubated at −20 °C for 5 min before adding 400 μL phenol–chloroform acid, subsequently vortexed for 1 min, and then centrifuged for 13 min at 12,000 RPM at room temperature in order to separate the mixture into aqueous and organic phases. This step was repeated when the interface was not compact. 

Furthermore, the aqueous (upper) phase was carefully removed without disturbing the lower phase and interphase and then transferred to a new tube. Considering the volume recovered, 80% isopropanol and 20% 3M sodium acetate pH 5.2 were added and homogenized. The sample was frozen overnight at −20 °C in an upright position, taking care not to mix during or after freezing. At the end of the incubation time, the mixture was centrifuged at 14,000 RPM for 10 min at room temperature, discarding the supernatant by inversion. Seventy-five percent ethanol (two volumes with respect to the amount of isopropanol/sodium acetate used) was added to the pellet and centrifuged at 10,000 RPM for 10 min at room temperature. The supernatant was discarded by inversion and the tubes were allowed to dry upside down for 10 min. Extracted RNA was resuspended with 50 uL of RNAsase-free water and then subjected to DNase I (catalog #EN0521, Thermo Fisher Scientific, Inc., USA) following the manufacturer’s instructions. Finally, the concentration and indexes of the readings obtained with the optical densities (OD) 260/230 and 260/280 were measured in a NanoDrop 2000 (Thermo Fisher Scientific, Inc., USA) and matched to the RNA quality indexes.

### 4.5. lncRNA Microarray Study

For lncRNA identification and differential expression analysis, the 30 samples (15 cases with AD and 15 healthy controls) were sent to Arraystar, Inc (Rockville, MD, USA), where RNA quality control and complementary RNA (cRNA) synthesis, labeling, and hybridization were performed according to Agilent’s single-color, microarray-based gene expression analysis protocol (Agilent Technologies, Santa Clara, CA, USA) with minor modifications.

#### 4.5.1. Quality Control

Before starting the microarray study, the quality, purity, and concentration of the exosomal RNA samples obtained were corroborated. To establish that the RNA is pure, the OD260/280 ratio should be close to 2.0, while the OD260/230 ratio must be >1.8. RNA integrity was assessed by denaturing agarose gel electrophoresis to detect clear bands of ribosomal RNA (rRNA) 28S and 18S with a 28S:18S intensity ratio close to 2:1, which indicates that RNA is intact.

#### 4.5.2. Complementary RNA Synthesis and Tagging

First, each sample was subjected to retrotranscription to obtain complementary DNA (cDNA); this was amplified and transcribed back to its complementary RNA (cRNA). In this step, amplification and incorporation of cyanine 3 (Cy3) fluorescent dye labeling can be achieved simultaneously along the entire length of the 3′ unbiased transcript using a random priming method (Arraystar Flash RNA Labelling Kit, Arraystar, Inc., Rockville, MD, USA). The labeled cRNAs were purified with the RNeasy mini kit (Qiagen, Hilden, Germany). In this step, it was possible to eliminate reagent residues and the excess of cyanine not incorporated. As a control of the amplification and labeling process of the samples, the concentration of the cRNA obtained and the rate of cyanine incorporation or specific activity (pmol of Cy3 per μg cRNA). Hybridization was allowed to continue if the cRNA concentration was >1.65 μg and the specific activity was >9 pmol of Cy3 per μg of cRNA. Otherwise, cRNA preparation was repeated.

#### 4.5.3. Hybridization and Microarray Scanning

A total of 1 μg of each labeled cRNA was fragmented by the addition of 5 μL of blocking agent 10× and 1 μL of fragmentation buffer 25×. The mixture was heated to 60 °C for 30 min, and then 25 μL of hybridization buffer 2× GE was added to dilute the labeled cRNA; 50 μL of hybridization solution was dispensed onto a hybridization plate, which was then assembled with a lncRNA expression microarray plate. The plates were incubated for 17 h at 65 °C in an Agilent hybridization oven. The hybridized arrays were washed and then scanned using an Agilent scanner (equipment #G2505C; Agilent Technologies, Santa Clara, CA USA).

#### 4.5.4. lncRNA Microarray and Data Normalization

Arraystar Human LncRNA Microarray v5.0 was used in this study. This microarray quantifies the expression of 39,317 lncRNAs (8393 gold-standard lncRNAs and 30,924 reliable lncRNAs). Arraystar, Inc has high-quality, proprietary lncRNA transcriptome databases, which compile lncRNAs through major public databases and repositories. Each transcript is identified with a splice probe. In this case, 60,491 probes of 60 nt length were used. For hybridization quality control, positive probes and negative probes for domestic genes, designed by the company, were used. Hybridization signals on the microarray were read using the Agilent’s Feature Extraction software, version 11.0.1.1. Data were normalized using quantile normalization [110] and expression values were adjusted using a linear model, as implemented in GeneSpring GX v12.1 (Agilent Technologies). 

#### 4.5.5. Identification of Differentially Expressed lncRNAs

After normalization, lncRNAs that were flagged as present or marginal (“all-target value”) in at least 15 out of 30 samples were chosen. Differentially expressed lncRNAs between cases and controls were determined based on the *p*-value of a two-sample *t*-test. To control false positives, these *p*-values were corrected using the false discovery rate (FDR) [111]. Thus, FDR-corrected *p*-values below a Type-I error of 5% (*P*_FDR_ < 0.05) were considered statistically significant. lncRNAs were also filtered using a Fold Change [FC] ≥1.5.

#### 4.5.6. lncRNA Annotation

Functional analysis of differentially expressed exosomal lncRNAs was performed using proprietary, high-quality transcriptome and lncRNA databases. Thus, information was obtained on lncRNA transcription unit identifications, including their length (number of nucleotides), gene identifications and symbols, *loci*, positions in relation to neighboring protein-coding genes (intronic or exonic overlap, sense, antisense, bidirectionality, or whether they are intergenic transcripts), functional molecular mechanisms, association with cells or tissues, and subcellular locations. In addition, scientific publications in the Web of Science, PubMed, and SCOPUS databases were continuously reviewed and selected, as well as the following databases for the annotation of results: LncRNAdb v2.0 (http://www.lncrnadb.org/), RNAdb v2.0 (http://research.imb.uq.edu.au/rnadb/), GENCODE v21 (https://www.gencodegenes.org), RefSeq (https://www.ncbi.nlm.nih.gov/refseq/), ENCODE CAGE (https://www.encodeproject.org), UCSC_knowngene (https://genome.ucsc.edu/cgi-bin/hgTrackUi?db=hg38&g=knownGene&c=chrX), FANTOM5CAT (https://fantom.gsc.riken.jp/5/data/), LncBook (https://ngdc.cncb.ac.cn/lncbook/), UCSC Genome Browser (https://genome.ucsc.edu/cgi-bin/hgGateway), MalaCards (https://www.malacards.org), GeneCards (https://www.genecards.org), KEGG (https://www.genome.jp/kegg/kegg2.html), STRING V11.5. (https://string-db.org/), Human Genome Connectome (HGC; https://hgc.rockefeller.edu/), and AlzGene (http://www.alzgene.org/). These websites were accessed between March and July 2023.

### 4.6. Statistical Analysis

A descriptive analysis was made of the sociodemographic variables according to their nature. For categorical variables such as gender, educational level, and marital status, frequencies and proportions were calculated and compared using a χ^2^-based test of independence. For continuous variables such as age at study entry, age at disease onset, weight, height, body mass index (BMI), and MMSE and MoCA test results, measures of central tendency and dispersion were calculated. Normality and homogeneity of variance were tested, respectively, with the Shapiro–Wilk and Bartlett tests. Continuous variables meeting the assumptions of normality and homogeneity of variance were compared using a two-sample *t*-test for independent samples and the nonparametric Wilcoxon test otherwise. Unless otherwise stated, all statistical analysis was performed in R version 4.3.1 [112].

### 4.7. ML-Based Predictive Model for AD Diagnosis Based on lnRNA Expression

A Machine Learning (ML)-based predictive model combining lncRNA expression with demographic variables such as gender, age, and educational level was designed and validated. For this purpose, several ML algorithms were explored, including Classification and Regression Trees (CART) [113], Random Forrest (RF) [30], Support Vector Machines (SVMs) [112,113], and eXtreme Gradient Boosting (XGBoost) [114]. A full list of ML algorithms is provided in Table S1 of the Supplementary Material.

ML-based predictive models were constructed and fitted using 70% of the data (21 individuals) as the training dataset and the remaining 30% of data (9 individuals) as the testing dataset. In all models, AD status (0: control; 1: case) was used as the dependent (outcome) variable and sex, years of education, and the expression levels of the identified lncRNAs were used as predictors. Due to the nature of the response variable, the parameters of the ML algorithms were determined as those that maximize the accuracy in predicting the diagnosis of AD. Subsequently, models were validated by calculating the measure of accuracy weighted on the test data, which expresses the percentage of individuals (case vs. control) correctly classified. Finally, the models were evaluated using ROC curve analysis and area under the ROC curve (AUC) as performance measures. Sensitivity, specificity, precision, positive predictive value (PPV), negative predictive value (NPV), and lift were measured in parallel. To identify the most important predictors of disease diagnosis, the relative importance of each of the variables included in the model was calculated as a measure reflecting the predictive power for AD diagnosis.

## 5. Conclusions

Alzheimer’s disease (AD) poses a significant public health challenge, being a leading cause of disability and dependency in the elderly, with profound physical, psychological, and economic impacts on caregivers, families, and society. Understanding the pathogenic mechanisms of AD is crucial for improving its management. While genetic risk variants have been a focus of many studies, there is growing recognition of the importance of elucidating the regulatory roles of genes, particularly non-coding RNAs (ncRNAs). These ncRNAs play key roles in modulating gene expression through intricate interactions with DNA, mRNA, and proteins, highlighting their potential significance in the development and progression of AD.

In this study, we examined the differential expression of long non-coding RNAs (lncRNAs) in circulating exosomes between individuals with Alzheimer’s disease (AD) and healthy controls. A total of 647 lncRNAs showed differential expression, with 550 being upregulated and 97 downregulated (Figure 1). These lncRNAs are implicated in gene expression regulation at various levels and are associated with functions such as chromatin modification, nuclear organization, and mRNA splicing, offering insights into AD pathology. Among the identified lncRNAs, 18 were found to potentially contribute to the AD pathogenic network (Table 5). In particular, the *TMEM186* and *AC109635.2* lncRNAs, targeting the *PMM2* and *SNX8*, respectively, are involved in Aβ peptide proteostasis. Additionally, *PROX1-AS*, targeting the *PROX1* gene, is linked to neurogenesis and cell differentiation processes. Finally, we explored the predictive capacity of demographic variables and expression levels of selected lncRNAs to diagnose AD in our population. For this purpose, 14 ML algorithms were implemented and evaluated. We were able to establish that the svmLinear2 ML algorithm was the most accurate, with an accuracy of >98% (Figure 3). Additionally, variable importance analyses revealed that the lncRNAs ENST00000608936 (*PROX1-AS1*) and ENST00000582092 (*SS18*) are the most relevant. Interestingly, using the OneR algorithm, we established that ENST00000608936 (*PROX1-AS1*) and ENST00000433747 (*AC073529.1*) show the highest relevance for AD diagnosis (Table 8 and Figure 6).

In the future, the differential expression of the lncRNAs identified in this study should be validated in independent cohorts. To this end, it is important to design a case–control study with a larger sample size than the one evaluated here and to use methods that allow the analysis of their differential expression, such as RT-PCRq. This validation would provide scientific support for the biological participation of these RNAs in the pathogenic network of AD. It is also necessary to perform in vitro and/or in vivo experimental validation of the results of the in silico analysis. With a view to achieving this objective, the regulation exerted by the lncRNAs identified on their respective associated target genes could be tested. With the scientific support that validates these results, therapeutic strategies aimed at the control of these associations could be developed for the redirection of their molecular functions. In this context, our work contributes significantly to understanding the molecular pathophysiology of AD, especially in this understudied population. The predictive model developed has the potential to be a valuable tool in clinical settings, enabling healthcare professionals to anticipate AD diagnosis. This, in turn, allows for the provision of preventive alternatives that can delay the onset and/or progression of the disease, thereby improving the quality of life for affected individuals and their caregivers.

## Figures and Tables

**Table 1:** Clinical and sociodemographic characterization of the study population.

Variable	All (*n* = 30)	Cases (*n* = 15)	Controls (*n* = 15)	*p*
	M (SD)			
Age (years)	79.8 (8.7)	77.5 (8.5)	82.1 (8.6)	0.261
Age of onset (years)	72.1 (7.2)	72.1 (7.2)	-	*-*
Weight (kg)	63.3 (15)	61.8 (15)	64.7 (15.4)	0.693
Height (m)	1.6 (0.07)	1.63 (0.08)	1.58 (0.06)	0.056
BMI (kg/m^2^)	24.5 (4.8)	23.1 (4.2)	25.9 (5.1)	0.161
MMSE	19.6 (9.6)	13.9 (9.5)	25.2 (5.6)	0.001
MoCA	15.3 (11.2)	5.5 (5.3)	25.9 (3)	<0.001
	**Frequency (%)**			
Sex				1
Female	22 (73.3%)	11 (73.3%)	11 (73.3%)	
Male	8 (26.7%)	4 (26.7%)	4 (26.7%)	
Education level				0.262
None	2 (13.3%)	2 (13.3%)	-	
Elementary	13 (43.3%)	7 (46.7%)	6 (40%)	
High school		-	-	
Tertiary	15 (50%)	6 (40%)	9 (60%)	
Postgraduate		-	-	
Marital status				0.753
Not reported		5 (33.3%)	7 (46.7%)	
Married		6 (40%)	5 (33.3%)	
Widowed		4 (26.7%)	3 (20%)	

SD: Standard deviation.

**Table 2:** Characteristics of the lncRNAs upregulated in the AD group.

Transcript	Gene ID	*P*	*P*_FDR_	FC	Length (nt)	Relationship to Nearest Coding Gene
ENST00000564869	*TMEM186*	7.7 × 10^−6^	0.229	1.78	561	Bidirectional
ENST00000608936	*PROX1-AS1*	2.6 × 10^−5^	0.234	1.73	900	Natural antisense
ENST00000529219	*AC109635.2*	5.5 × 10^−5^	0.234	1.55	500	Intergenic
ENST00000419745	*LINC02043*	6.9 × 10^−5^	0.234	1.55	1832	Intergenic
ENST00000589662	*AC022031.2*	8.2 × 10^−5^	0.234	3.26	513	Intergenic
ENST00000641383	*AP003175.1*	8.7 × 10^−5^	0.234	2.00	6658	Intergenic
ENST00000420172	*AL020993.1*	9.2 × 10^−5^	0.234	1.86	297	Natural antisense
TCONS_00024747	*XLOC_012031*	1.1 × 10^−4^	0.234	1.73	880	Intergenic
ENCT00000179246	*CATG00000032665.1*	1.1 × 10^−4^	0.234	1.51	1963	Natural antisense
ENST00000586145	*LINC01255*	1.4 × 10^−4^	0.253	1.82	463	Intergenic
ENST00000433746	*ERICH3*	1.4 × 10^−4^	0.253	1.52	5076	Sense with overlap in exon
uc002dam.1	*DEXI*	1.5 × 10^−4^	0.253	1.59	2128	Bidirectional
ENST00000582092	*SS18*	1.7 × 10^−4^	0.253	1.71	215	Sense with overlap in exon
ENST00000417218	*AC244021.1*	1.8 × 10^−4^	0.253	1.76	3662	Intergenic
ENST00000530595	*AP000662.1*	1.9 × 10^−4^	0.253	2.07	2790	Bidirectional
ENST00000420563	*AC053503.2*	2.0 × 10^−4^	0.253	1.70	1598	Bidirectional
ENST00000648691	*AC020907.1*	2.4 × 10^−4^	0.253	1.97	1429	Intergenic
TCONS_00013683	*XLOC_006345*	2.6 × 10^−4^	0.253	1.98	292	Intergenic
ENST00000515186	*AC105345.2*	2.6 × 10^−4^	0.253	2.07	664	Natural antisense
ENST00000522704	*AC091939.1*	2.6 × 10^−4^	0.253	2.57	691	Natural antisense
ENST00000602357	*ASMTL-AS1*	2.6 × 10^−4^	0.253	2.06	435	Natural antisense
ENST00000535567	*FAM66C*	2.7 × 10^−4^	0.253	1.55	724	Natural antisense
T081902	*G018952*	2.8 × 10^−4^	0.253	1.76	6159	Intergenic
ENST00000608506	*LINC00635*	2.8 × 10^−4^	0.253	1.76	482	Intergenic

**Table 3:** Characteristics of the lncRNAs downregulated in the AD group.

Transcript	Gene ID	*P*	*P*_FDR_	FC	Length (nt)	Relationship to Nearest Coding Gene
ENST00000433747	*AC073529.1*	2.6 × 10^−4^	0.253	1.54	1071	Intergenic
ENST00000507461	*C5orf64*	4.6 × 10^−4^	0.312	1.52	535	Intergenic
T380457	*G090124*	2.6 × 10^−3^	0.473	1.77	2072	Intergenic
ENST00000637865	*TAB2-AS1*	4.5 × 10^−3^	0.541	1.50	794	Intronic antisense
ENST00000564748	*AC117382.2*	4.6 × 10^−3^	0.541	1.58	3151	Intergenic
T064254	*G014791*	4.7 × 10^−3^	0.541	1.59	685	Intergenic
ENST00000565073	*AC007342.1*	6.2 × 10^−3^	0.561	1.53	706	Sense with overlap in intron
ENST00000452352	*HTR2A-AS1*	6.4 × 10^−3^	0.561	1.55	384	Intronic antisense
ENST00000476783	*PTBP2*	6.9 × 10^−3^	0.578	1.59	779	Sense with overlapping in exon
ENST00000519833	*IL7*	7.0 × 10^−3^	0.578	1.61	721	Bidirectional
ENST00000626729	*LINC01232*	7.3 × 10^−3^	0.578	1.77	2796	Intronic antisense
ENST00000414600	*SOX9-AS1*	7.5 × 10^−3^	0.582	2.91	742	Natural antisense
ENST00000591283	*AC021683.2*	8.1 × 10^−3^	0.601	1.54	4902	Intergenic
ENST00000645809	*PCA3*	8.2 × 10^−3^	0.601	1.95	3735	Intronic antisense
NR_026562	*C20orf24*	1.0 × 10^−2^	0.628	2.15	1251	Bidirectional
ENST00000441809	*AL359771.1*	1.1 × 10^−2^	0.637	1.52	379	Intergenic
ENST00000650187	*AL132996.1*	1.1 × 10^−2^	0.642	1.59	1271	Intergenic
ENST00000553299	*GNG2*	1.2 × 10^−2^	0.642	1.51	1542	Sense with overlapping in exon
TCONS_00008636	*XLOC_004141*	1.3 × 10^−2^	0.652	1.51	231	Intergenic
T185193	*G042518*	1.5 × 10^−2^	0.685	1.55	4574	Intergenic
ENST00000512359	*HHIP-AS1*	1.5 × 10^−2^	0.690	1.52	646	Natural antisense
T287007	*G066968*	1.5 × 10^−2^	0.690	2.49	9295	Intergenic
ENST00000585367	*HSD52*	1.6 × 10^−2^	0.701	1.58	401	Intergenic
ENST00000503266	*LINC01365*	1.6 × 10^−2^	0.701	1.69	550	Intergenic
T215907	*G049958*	1.7 × 10^−2^	0.711	2.73	6899	Natural antisense

**Table 4:** Possible lncRNA-associated genes identified among the study groups.

Transcript ID	Gene ID	Expression in AD Cases	Associated Transcription	Gene ID	Type	Locus	Strand	Number of Base-Pairing Interactions Predicted by RIblast [29]	Sum of Local Energies of Base-Pairing Interaction (kcal/mol)
ENST00000564869	*TMEM186*	Upregulated	ENST00000566983	*PMM2*	mRNA	chr16:8882680-8941725	+		
ENST00000608936	*PROX1-AS1*	Upregulated	ENST00000471129	*PROX1*	mRNA	chr1:213983181-213997225	+		
ENST00000529219	*AC109635.2*	Upregulated	ENST00000222990	*SNX8*	mRNA	chr7:2251770-2314464	-	53	−1068.97
ENST00000419745	*LINC02043*	Upregulated	ENST00000262160	*SMAD2*	mRNA	chr18:47808957-47930559	-	87	−3068.26
ENST00000589662	*AC022031.2*	Upregulated	ENSG00000233695	*GAS6-AS1*	lncRNA	chr13:113815609-113845746	-	40	−769.8
ENST00000453342	*POT1-AS1*	Upregulated	ENST00000357628	*POT1*	mRNA	chr7:124822386-124929981	-	1	−52.31
ENST00000420172	*AL020993.1*	Upregulated	ENST00000617146	*SRCIN1*	mRNA	chr17:38530016-38605930	-	1	−16.07
ENST00000433746	*ERICH3*	Upregulated	ENST00000326665	*ERICH3*	mRNA	chr1:74568123-74673792			
uc002dam.1	*DEXI*	Upregulated	ENST00000646979	*CIITA*	mRNA	chr16:10922167-10936388			
ENST00000582092	*SS18*	Upregulated	ENST00000415083	*SS18*	mRNA	chr18:26016253-26090613			
ENST00000433747	*AC073529.1*	Downregulated	ENST00000295851	*ABI2*	mRNA	chr2:203328219-203447723	+	6	−104.58
ENST00000637865	*TAB2-AS1*	Downregulated	ENST00000606202	*TAB2*	mRNA	Chr6:149539777-149699665	+		
ENST00000564748	*AC117382.2*	Downregulated	ENST00000378505	*RPGR*	mRNA	chrX:38284409-38327544	-	350	−10,193.80
ENST00000565073	*AC007342.1*	Downregulated	NM_001323610	*RBL2*	mRNA	Chr16:53433977-53491648	+		
ENST00000452352	*HTR2A-AS1*	Downregulated	ENST00000378688	*HTR2A*	mRNA	Chr13:46831546-46897076	-		
ENST00000626729	*LINC01232*	Downregulated	ENST00000361558	*MUC2*	mRNA	chr11:1074875-1110511	+	223	−4588.2
ENST00000414600	*SOX9-AS1*	Downregulated	ENST00000645356	*SOX9*	mRNA	Chr17:72121020-72126416	+		
ENST00000645809	*PCA3*	Downregulated	ENST00000428286	*PRUNE2*	mRNA	chr9:76611377-76906087	-		

**Table 5:** Role of exosomal lncRNAs differentially expressed in AD pathogenicity.

lncRNA (This Study)	Target Gene	AD Pathogenic Network
*TMEM186*	*PMM2*	Proteostasis of Aβ-peptide
*PROX1-AS1*	*PROX1*	Neurogenesis and cell differentiation
*AC109635.2*	*SNX8*	Proteostasis of Aβ-peptide
*LINC02043*	*SMAD2*	Neurogenesis and cell differentiation, proteostasis of Aβ-peptide, and neuroinflammation
*AC022031.2*	GAS6-AS1 *	Neurogenesis and cell differentiation, proteostasis of Aβ-peptide, neuroinflammation, and apoptosis
*POT1-AS1*	*POT1*	Proteostasis of the phosphorylated Tau protein
*AL020993.1*	*SRCIN1*	Neurite growth and synaptic plasticity
*ERICH3*	*ERICH3*	Negative response to treatment of depression with SSRIs
*DEXI*	*CIITA*	Neuroinflammation
*SS18*	*SS18*	Chromatin remodeling
*AC073529.1*	*ABI2*	Neurite growth
*TAB2-AS1*	*TAB2*	Proteostasis of Aβ-peptide, neuroinflammation, and apoptosis
*AC117382.2*	*RPGR*	Ciliopathies
*AC007342.1*	*RBL2*	Cell cycle control
*HTR2A-AS1*	*HTR2A*	Depression
*LINC01232*	*MUC2*	Alteration of the intestinal barrier
*SOX9-AS1*	*SOX9*	Proteostasis of Aβ-peptide, neuroinflammation, and apoptosis
*PCA3*	*PRUN2*	Apoptosis

* Interacts with *GAS6.*

**Table 6:** PPI identified using the 18 lncRNA-associated genes.

Protein	ACC	*P*_FDR_
PMM2	0.885	3.27 × 10^−11^
PROX1	0.879	0.00216
SNX8	0.982	5.55 × 10^−16^
SMAD2	0.841	1.08 × 10^−5^
GAS6	0.719	6.45 × 10^−7^
POT1	1.000	<1.0 × 10^−16^
SRCIN1	0.772	0.00376
CIITA	0.900	1.41 × 10^−8^
SS18	1.000	<1.0 × 10^−16^
ABI2	1.000	<1.0 × 10^−16^
TAB2	0.923	7.16 × 10^−9^
RPGR	0.899	1.14 × 10^−14^
RBL2	0.968	1.11 × 10^−16^
HTR2A	0.824	4.42 × 10^−8^
SOX9	0.799	1.64 × 10^−5^

ACC: Average clustering coefficient. FDR: False discovery rate.

**Table 7:** Biological distance between lncRNA-associated genes and previously reported AD genes.

Gene ID (This Study)	AD Gene	Distance	*P*	*P*_FDR_
*GAS6*	*APOE*	4.72	0.036	0.352
	*CLU*	1.11	0.001	0.098
	*ABCA7*	10.00	0.041	0.352
	*CD2AP*	4.44	0.012	0.332
*SMAD2*	*APOE*	4.44	0.004	0.274
	*CLU*	4.44	0.008	0.274
	*BIN1*	4.72	0.015	0.333
	*CD33*	5.69	0.023	0.352
	*ABCA7*	10.00	0.023	0.352
*HTR2A*	*CLU*	4.44	0.017	0.337
	*ABCA7*	10.00	0.034	0.352
*RBL2*	*CD33*	5.69	0.037	0.352
*CIITA*	*CD33*	4.72	0.008	0.274
*MUC2*	*CR1*	5.00	0.037	0.352
*SRCIN1*	*CD2AP*	6.03	0.048	0.404
*SS18*	*BIN1*	5.97	0.029	0.352
*POT1*	*PICALM*	8.63	0.048	0.352

**Table 8:** Accuracy of the OneR ML algorithm when each selected lncRNA is used for predicting AD diagnosis.

lncRNA	Accuracy
*PROX1-AS1* (ENST00000608936)	86.36%
*AC022031.2* (ENST00000589662)	86.36%
*POT1-AS1* (ENST00000453342)	86.36%
*TMEM186* (ENST00000564869)	81.82%
*ERICH3* (ENST00000433746)	81.82%
*SS18* (ENST00000582092)	81.82%
*AC073529.1* (ENST00000433747)	81.82%
*AC109635.2* (ENST00000529219)	77.27%
*AL020993.1* (ENST00000420172)	77.27%
*DEXI* (uc002dam.1)	77.27%
*PCA3* (ENST00000645809)	77.27%
*LINC02043* (ENST00000419745)	72.73%
*AC117382.2* (ENST00000564748)	72.73%
*AC007342.1* (ENST00000565073)	72.73%
*SOX9-AS1* (ENST00000414600)	72.73%
*HTR2A-AS1* (ENST00000452352)	63.64%
*LINC01232* (ENST00000626729)	63.64%
*TAB2-AS1* (ENST00000637865)	59.09%

## Data Availability

The data presented in this study are available on reasonable request from the corresponding authors. The data are not publicly available due to the ongoing nature of the study and our commitment to protecting the privacy and confidentiality of our patients.

## Supplementary Materials

The following supporting information can be downloaded at: https://www.mdpi.com/article/10.3390/ijms25147641/s1, [115,116,117,118].
