# Supplementary material for: Long Non-Coding RNAs and Alzheimer’s Disease: Towards Personalized Diagnosis
Source: Int J Mol Sci. 2024 Jul 11;25(14):7641. doi: 10.3390/ijms25147641 (PMC11277322; doi:10.3390/ijms25147641)
Supplement: Supplementary file 1 [file ijms-25-07641-s001.zip › ijms-3050914-supplementary.pdf]

International Journal of
*Molecular Sciences*

MDPI

*Supplementary Materials*

# Long Non-Coding RNAs and Alzheimer's Disease: Towards Personalized Diagnosis

**Maria I. Mosquera-Heredia [1], Oscar M. Vidal [1], Luis C. Morales [1], Carlos Silvera-Redondo [1], Ernesto Barceló [2,3,4], Ricardo Allegri [5], Mauricio Arcos-Burgos [6], Jorge I. Vélez [7,*,†] and Pilar Garavito-Galofre [1,*,†]**

[1] Department of Medicine, Universidad del Norte, Barranquilla 081007, Colombia; mosquerai@uninorte.edu.co (M.I.M.-H.); oorjuela@uninorte.edu.co (O.M.V.); burbanoc@uninorte.edu.co (L.C.M.); csilvera@uninorte.edu.co (C.S.-R.)
[2] Instituto Colombiano de Neuropedagogía, Barranquilla 080020, Colombia; erbarcelo@yahoo.com
[3] Department of Health Sciences, Universidad de La Costa, Barranquilla 080002, Colombia
[4] Grupo Internacional de Investigación Neuro-Conductual (GIINCO), Universidad de La Costa, Barranquilla 080002, Colombia
[5] Institute for Neurological Research FLENI, Montañeses 2325, Buenos Aires C1428AQK, Argentina; rallegri@fleni.org.ar
[6] Grupo de Investigación en Psiquiatría (GIPSI), Departamento de Psiquiatría, Instituto de Investigaciones Médicas, Facultad de Medicina, Universidad de Antioquia, Medellin 050010, Colombia; mauricio.arcos@udea.edu.co
[7] Department of Industrial Engineering, Universidad del Norte, Barranquilla 081007, Colombia
* Correspondence: jvelezv@uninorte.edu.co (J.I.V.); mpgaravi@uninorte.edu.co (P.G.-G.)
† These authors contributed equally to this work.

International Journal of
*Molecular Sciences*

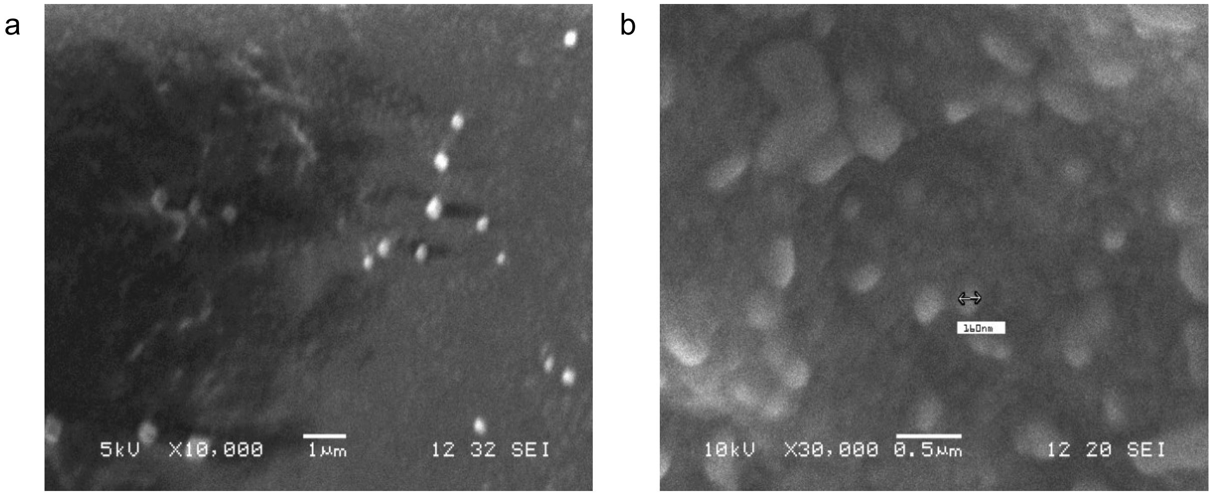


**Figure S1.** Results of the Scanning Electron Microscopy (SEM) for characterizing exosomes. For this purpose, **(a)** exosomes were encapsulated with nanodiamond particles, and **(b)** their sizes were meticulously confirmed, with measurements revealing a maximum diameter of 160 nm.

**Machine Learning algorithms for Alzheimer's disease diagnosis prediction**

We used Machine Learning (ML) algorithms to construct predictive models of Alzheimer's disease (AD) diagnosis based on long non-coding RNA (lncRNA) signatures in individuals from Barranquilla, Colombia. Models were implemented in the caret package (Kunh, 2020) in R (https://www.R-project.org/). The complete list of ML algorithms implemented in caret is available at https://topepo.github.io/caret/available-models.html.

Table S1 shows the ML algorithms used in this study. In all cases, the set of predictors for AD diagnosis consisted of demographic variables, and lncRNA expression. See the Methods section in the main manuscript for more details.

**Table S1.** ML algorithms used to predict AD diagnosis.

| Abbreviation | Model | Tuning parameters |
|---|---|---|
| avNNet | Model Averaged Neural Network | size, decay, bag |
| knn | k-Nearest Neighbours | k |
| lda | Linear discriminant analysis | None |
| rf | Random Forest | mtry |
| rpart | CART | cp |
| rpart1SE | CART | None |
| rpart2 | CART | maxdepth |
| svmLinear | SVM with Linear Kernel | C |
| svmLinear2 | SVM with Linear Kernel | Cost |
| svmPoly | SVM with Polynomial kernel | degree, scale, C |
| svmRadial | SVM with Radial Basis Function Kernel | sigma, C |
| treebag | Bagged CART | None |
| xgbLinear | eXtreme Gradient Boosting (XGBoost) | nrounds, lambda, alpha, eta |
| xgbTree | eXtreme Gradient Boosting Tree | nrounds, max_depth, eta, gamma, colsample_bytree, min_child_weight, subsample |

CART: Classification and Regression Tree (CART)(L. Breiman, Friedman, Olshen, & Stone, 1984); RF: Random Forest (RF)(L. Breiman, 2001; Satterfield, Cantwell, & Satterfield); SVM: Support Vector Machine (SVM)(Cortes & Vapnik, 1995; Salazar, Vélez, & Salazar, 2012); XGBoost: eXtreme Gradient Boosting (Chen & Guestrin, 2016; Chen et al., 2020).

**References**


1. Breiman, L. (2001). Random Forests. In R. E. Schapire (Ed.), *Machine Learning* (Vol. 45, pp. 5-32). Statistics Department, University of California, Berkeley, CA 94720: Kluwer Academic Publishers. Manufactured in The Netherlands.
2. Breiman, L., Friedman, J. H., Olshen, R. A., & Stone, C. H. (1984). *Classification and Regression Trees.* Belmont, CA: Wadsworth International Group, Inc.
3. Chen, T., & Guestrin, C. (2016). *XGBoost: A Scalable Tree Boosting System*. Paper presented at the 22nd SIGKDD Conference on Knowledge Discovery and Data Mining. https://arxiv.org/abs/1603.02754
4. Chen, T., He, T., Benesty, M., Khotilovich, V., Tang, Y., Cho, H., . . . Li, Y. (2020). xgboost: Extreme Gradient Boosting. R package version 1.0.0.2. URL: https://CRAN.R-project.org/package=xgboost.
5. Cortes, C., & Vapnik, V. (1995). Support-vector networks. *Machine Learning, 20*, 273-297. doi:https://doi.org/10.1007/BF00994018
6. Kunh, M. (2020). caret: Classification and Regression Training. (Version R package version 6.0-86). Retrieved from https://CRAN.R-project.org/package=caret
7. Salazar, D. A., Vélez, J. I., & Salazar, J. C. (2012). Comparison between SVM and Logistic Regression: Which one is Better to Discriminate? *Revista Colombiana de Estadistica, 35*(2), 223-237.
8. Satterfield, J. H., Cantwell, D. P., & Satterfield, B. T. (1974). Pathophysiology of the hyperactive child syndrome. *Arch Gen Psychiatry, 31*(6), 839-844. Retrieved from https://www.ncbi.nlm.nih.gov/pubmed/4441251
